# The Role of Resveratrol in Eye Diseases—A Review of the Literature

**DOI:** 10.3390/nu14142974

**Published:** 2022-07-20

**Authors:** Anna Bryl, Mariusz Falkowski, Katarzyna Zorena, Małgorzata Mrugacz

**Affiliations:** 1 Department of Ophthalmology and Eye Rehabilitation, Medical University of Bialystok, 15-089 Bialystok, Poland; malgorzata.mrugacz@umb.edu.pl; 2 PhD Studies, Medical University of Bialystok, 15-089 Bialystok, Poland; mariusz.falkowski@adres.pl; 3 Department of Immunobiology and Environmental Microbiology, Medical University of Gdansk, 80-211 Gdansk, Poland; katarzyna.zorena@gumed.edu.pl

**Keywords:** resveratrol, molecular mechanisms, chronic eye diseases

## Abstract

Resveratrol (3,5,4′-trans-trihydroxystilbene) is a polyphenolic phytoalexin belonging to the stilbene family. It is commonly found in grape skins and seeds, as well as other plant-based foods. Oxidative stress and inflammation play a key role in the initiation and progression of age-related eye disorders (glaucoma, cataracts, diabetic retinopathy, and macular degeneration) that lead to a progressive loss of vision and blindness. Even though the way resveratrol affects the human body and the course of many diseases is still the subject of ongoing scientific research, it has been shown that the broad spectrum of anti-inflammatory and neuroprotective properties of resveratrol has a beneficial effect on eye tissues. In our research, we decided to analyze the current scientific literature on resveratrol, its possible mechanisms of action, and its therapeutic application in order to assess its effectiveness in eye diseases.

## 1. Introduction

Resveratrol (3,5,4′-trans-trihydroxystilbene), which belongs to the stilbene family, is a polyphenolic phytoalexin. It is mainly found in grape skins and seeds but also in peanuts, berries, and tea [1]. It is a component of red wine [2]. It is synthesized by numerous plant species in response to injury, stress, infections, and UV radiation [3]. Resveratrol (RSV) was isolated and described for the first time in 1939 [4]. Interest in RSV among scientists increased significantly when epidemiologists announced in 1981 that the French had a lower coronary heart disease mortality despite risk-increasing habits (smoking and eating high amounts of saturated fats) [5,6]. This phenomenon was explained by moderate consumption of red wine [7]. Resveratrol may play a role in modulating a variety of intracellular enzymes, such as kinases, lipoxygenases, cyclooxygenases, and free radical scavengers [8]. As a result, it exhibits a wide range of biological properties, among others, anti-glycation, antioxidant, anti-inflammatory, neuroprotective, and antineoplastic effects [9]. However, due to its low bioavailability and quick metabolism, the possibility of utilizing its properties is limited, which makes RSV an ongoing subject of numerous clinical trials.

In our research, we decided to analyze the current scientific literature on resveratrol, its possible mechanisms of action, and its therapeutic application in order to assess its effectiveness in eye diseases. Peer-reviewed journals were the source of all articles we had considered, and pertinent articles have been found in ScienceDirect, Web of Science, and PubMed databases. The following keywords have been used in our search: “resveratrol”, “transsveratrol”, and “molecular mechanism of resveratrol.” Each of these keywords was used in conjunction with words such as “eye”, “chronic eye disease”, and “retina” in order to refine the search. After reviewing the available literature, we have included relevant information on RSV and its impact on the eyes in our work.

## 2. Resveratrol and Its Properties

**Figure 1:**
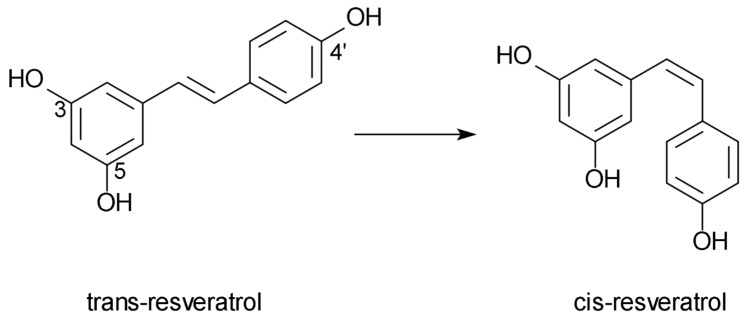
The chemical formula of trans- and cis-resveratrol.

RSV is stilbene in structure which consists of two aromatic rings connected by a methylene bridge. Two forms of RSV exist—cis- and trans-resveratrol. The form of RSV most commonly found in grapes and grape juice is trans-resveratrol-3-O-β-glucoside (trans-piceid) [10]. The cis-RSV form is formed due to light affecting the trans-form [11]. The chemical formulas of trans- and cis-RSV are shown in Figure 1 [12].

The molecular properties of RSV, which were first described in 2003, are determined by its chemical structure [6]. RSV can modulate SIRT1 activity, which influences the acetylation status of p53 and DNA repair enzymes [13]. The binding of RSV to the *N*-terminal domain of SIRT1 strengthens the bonds between SIRT and the p53 peptide, thus increasing the activity of SIRT1 [6,13]. It has been determined that there is a possibility for the tyrosine-like phenolic ring of RSV to match into the active site pocket of tyrosyl transfer RNA (tRNA) synthetase. This has an effect on the downstream activation of key stress signaling pathways [6,14]. It has also been demonstrated that RSV acts as a PPAR antagonist through its direct interaction with PPARγ and PPARα and is a ligand for leukotriene A4 hydrolase (an enzyme associated with the metabolism of arachidonic acid) [15,16].

After oral administration, RSV is absorbed by enterocytes [17]. However, only a negligible amount reaches the bloodstream and body tissues [18]. Oral bioavailability accounts for approximately 12% of transsveratrol [19]. The processes of passive diffusion or carrier-mediated transport across the enterocyte apical membrane help absorb RSV, and they quickly and extensively metabolize it to resveratrol glucuronides or sulfates [20]. A total of 90% of the ingested RSV reaches the colon intact and is then subjected to enteric fermentation. The polyphenolic metabolites are absorbed via the portal vein and then travel to the liver, where they are further methylated, glucuronidated or sulfated. In the next step, the metabolites penetrate into the systematic circulation and finally reach the tissues and cells where they exhibit physiological significance [21]. Resveratrol and unused metabolites return to the small intestine and are cleared in the bile or excreted in the urine [18,21].

### 2.1. Anti-Inflammatory Properties

Inflammation results from the secretion of chemokines/cytokines (also known as interleukins) [22]. It has been shown that RSV inhibits the transcription and translation of IL-6 (interleukin 6), which results in its reduced secretion by macrophages [23]. Similarly, when RSV is administered to monocyte cultures, a reduction in the expression of inflammatory mediators is observed: TNF-α (tumor necrosis factor α) and IL-8 (interleukin 8) [24]. What is more, resveratrol is involved in the inhibition of toll-like receptors. Pro-inflammatory cytokines and chemokine expression may be induced by these receptors. Moreover, the activation of innate and adaptive immunity is stimulated by them [9,25]. Depending on the dose, RSV reduces the expression of matrix metalloprotease and inhibits the production of IL-1 (interleukin 1), IL-6, and TNF-α [26]. In addition, it was found that resveratrol is effective in suppressing NF-κB (nuclear factor-κB) signaling due to inhibiting the activity of NF-κB and IκB kinase and suppressing the phosphorylation of JAK/STAT signaling pathways [27]. RSV inhibits the pro-inflammatory gene expression in human monocytes by stimulating the synthesis of anti-inflammatory microRNAs [28]. Numerous polyphenols inhibit the expression of COX (cyclooxygenase) genes. It has been proven that resveratrol inhibits the action of COX-1 and COX-2 in a dose-dependent manner [20,29,30].

Resveratrol reportedly regulates inflammatory responses via various signaling pathways. These include the AA pathway, nuclear factor kappa B (NF-κb), Mitogen-activated protein kinase (MAPK), and activator protein-1 (AP-1) [21].

#### 2.1.1. Arachidonic Acid (AA) Pathway

Hamowanie szlaku AA pathway inhibition plays an important role [31,32]–membrane phospholipids release AA with the cleavage of phospholipase A2. COX metabolizes AA with prostaglandin (such as PGD2, PGE2, PGI2) and thromboxane (TX) A2 [33] generation. COX (COX-1 and COX-2) are important sources of PG [34,35]. Prostanoids produced via COX-1 exert renal homeostasis, cytoprotective, immunomodulatory, and platelet function [36], while those derived from COX-2 participate in the inflammatory response [37]. Jang et al. found that this polyphenol was able to selectively suppress the COX activity of COX-1 and this isoenzyme’s hydroperoxidase activity, thus inhibiting PG synthesis [38].

#### 2.1.2. NF-κB Pathway

A wide assortment of genes expression regulating inflammatory responses is modulated by NF-κB. NF-κB1 (p50/p105), NF-κB2 (p52/p100), p65 (RelA), RelB, and c-Rel are all in the family of the NF-κB/Rel transcription factor [39]. NF-κB is usually found in an inactive form within the cytoplasm. Its interaction is observed with κB (IκB) inhibitors, such as IκBα and IκBβ. The IKK-mediated phosphorylation of IκB constitutes an important step in the activation of NF-kB [40]. Two pathways for NF-kB activation exist: the classical pathway and the alternative pathway [41,42]. Inflammatory cytokines (IL-1, IL-6, IL-10, and TNF) in LPS stimulating cells (lipopolysaccharide) may be expressed due to activation of NF-kB [43]. The ability of RSV to inhibit NO synthase (NOS) and down-regulate NF-kB activation in macrophages is high [44]. Moreover, inhibition of NF-κB activation and expression of NF-κB regulated genes by RSV is associated with suppression of IκB kinase activation. TLR-4 expression is diminished by RSV. Conversely, RSV induces the LPS-induced development of IL-6, NO, and iNOS, inhibits IκBα phosphorylation, and prevents NF-κB p65 translocation to the nucleus from the cytoplasm [21,39]. RSV exhibits anti-inflammatory and immunomodulatory functions through activation of sirtuin-1 (Sirt-1). As a deacetylase, Sirt-1 plays an important role in immune tolerance and blocks the TLR-4/NF-κB/STAT pathway with reduced inflammatory factors [21,39].

#### 2.1.3. MAPK (Mitogen-Activated Protein Kinases) Pathway

MAPKs play a key role in many biological processes, including proliferation, differentiation, apoptosis, inflammation, and environmental stress responses [45]. MAPK makes up a group of kinases, including c-jun *N*-terminal kinase (JNK), extracellular signal-regulated kinase (ERK), Big MAP kinase (BMK), and p38 [46,47]. p38 MAPK is activated by some pro-inflammatory stimuli such as oxidative stress, UBV, and inflammatory cytokines [48,49]. RSV has been reported to inhibit COX-2 expression in ex vivo mouse skin due to inhibiting PMA-induced activation of ERK and p38 MAPK pathways [50]. The inhibitory effect of this compound may involve the inhibition of the p38 MAPK-cytosolic phospholipase A2-AA-TxA2- [Ca^2+^] cascade and activation of NO/cyclic GMP and then suppression of phospholipase C and/or PKC activation [51]. RSV allows for the suppression of the inflammatory response by blocking p65 and IκB phosphorylation protein expression from NF-κB signaling and p38 and ERK phosphorylation from MAPK signaling [52].

### 2.2. Anti-Glycation Properties

A reaction that occurs between proteins and reduces sugars is known as glycation. It results in the formation of advanced glycation end products (AGEs). AGEs generate damage at the level of tissues and cells. Endothelial dysfunction, changes in the structure of proteins, lipid peroxidation, and stimulation of inappropriate cellular activity are also the possible effects of AGEs [9]. Glycation produces highly reactive dicarbonyl compounds such as methylglyoxal (MGO) and glyoxal (GO). These are key precursors for AGE formation [9]. MGO and GO increase oxidative stress [9]. It has been shown that polyphenols, including resveratrol, exhibit anti-glycation properties, inhibit AGE formation and bind to MGO and GO [9,53,54,55,56]. Yılmaz et al. demonstrated that the administration of drinking water with resveratrol to rats considerably reduced the levels of advanced oxidation protein products (AOPP), AGE, and protein carbonyl in the plasma and oxidative stress markers in the liver [57].

### 2.3. Antioxidant Properties

Oxidative stress may be understood as an imbalance between the generation of reactive oxygen species (ROS) and the oxidant production favored by the antioxidant defense. Macromolecules are damaged, and their functions are impaired by oxidative stress. Numerous diseases related to age, such as chronic kidney disease, cancer, diabetes, cardiovascular and neurodegenerative diseases, can be attributed to that [9,58]. Three hydroxyl groups drive the action of resveratrol and make it a powerful antioxidant. It has an inhibitory effect on ROS production, abnormal mitochondrial distribution, and lipid peroxidation [55,58,59]. Moreover, the presence of RSV in epithelial cells protects them against oxidative damage caused by hydrogen peroxide and boosts the expression and phosphorylation of occludin as well as other zonula occludin proteins [59]. Administering resveratrol to rats suffering from diabetes (5 mg/kg/day) results in the normalization of their antioxidant status [60]. However, resveratrol administration at doses of 10 or 20 mg/kg of body mass for 4 weeks in rats suffering from streptozotocin-induced diabetes leads to a reduction of AOPP, as well as the activity of catalase (CAT) and superoxide dismutase (SOD) in the lens [61]. Animals treated with resveratrol exhibit decreased ROS production, increased membrane potential, and cytochrome c release inhibition from the inner mitochondrial membrane [9,62].

RSV acts as an antioxidant by controlling antioxidant enzymes and blocking free radical damage to DNA. The level of 8-oxo-7,8-dihydroxy-29-deoxyguanosine, a known marker of oxidative DNA damage, decreases with the addition of resveratrol in vivo [63]. RSV may protect IPEC-J2 cells from oxidative damage by stimulating the Nrf2 pathway [64]. RSV increases the expression of the Nrf2 and HO-1 signaling pathway proteins and thus attenuates oxidative stress and hypoxia-induced and chemically induced inflammatory response in neonatal rats [65]. RSV also inhibits MAPK activation and reduces the production of inflammatory mediators by activating the SIRT1/AMPK and Nrf2 antioxidant defense pathways [66].

### 2.4. Neuroprotective Properties

RSV reduces neuronal damage and improves the functioning of the central nervous system [9]. It reduces cerebral cortex neurodegeneration and improves memory recovery in mice following fluoride [1]. It also improves cognition, learning, and memory in rats suffering from vascular dementia [67]. Its neuroprotective properties have been demonstrated in cerebral ischemia or intracerebral hemorrhages [68]. Moreover, at doses of 10–80 mg/kg daily, RSV may constitute an effective method of treating depression [69].

Van et al. studied the neuroprotective effect of RSV on experimental ischaemic damage to the retina. In this study, RSV or saline was administered to adult rats by intraperitoneal injection for 5 days on a daily basis. On the third day, the rats were induced with ischaemic damage to the retina by increasing intraocular pressure for 45 min. Retinal function was measured 1 week after the ischaemic injury and compared with the results prior to the administration of RSV. Ischaemic injury caused visible thinning of the inner retinal layers, but this was reduced in the rats receiving resveratrol [70]. A study on the possible neuroprotective action of RSV against photoreceptor cell death in a rodent model of retinal detachment found that resveratrol blocks Caspase 3, 8, and 9 activation and upregulates the FoxO family. It has been concluded that the role of resveratrol in preventing vision loss in diseases by photoreceptor detachment may be present [71].

### 2.5. Antineoplastic Properties

Resveratrol has been shown to have antineoplastic properties [72,73,74,75,76]. Systemic administration of RSV inhibits tumor initiation and growth through a variety of mechanisms, among them cell cycle arrest, apoptosis induction, and inhibition of angiogenesis [11]. It protects healthy cells while inducing death in cancer cells. The dual mode of action of RSV is dose-dependent. Whether the cell is healthy or pathological, lower concentrations increase the expression of cell survival proteins, while higher doses induce cell apoptosis or necrosis [9,77,78]. In high doses, RSV inhibits the synthesis of nucleic acids and proteins and causes chromatin structure impairment and eventually cell death [79].

### 2.6. Vasorelaxant Properities

RSV may induce vasodilation [80]. It may therefore be a candidate for the treatment of eye disorders associated with impaired perfusion [81]. Vasorelaxation of small-diameter retinal arterioles was observed in a dose-dependent manner when treated with resveratrol [82]. Nagaoka et al. blocked the synthesis of other molecules involved in vasodilation, particularly prostacyclin and cytochrome P450 metabolites [82]. This had no effect on the RSV response. In their study, they have provided evidence of two independent mechanisms by which RSV caused vasodilatation and, therefore, vasodilation in isolated retinal arterioles [81,82].

## 3. Resveratrol in Eye Diseases

### 3.1. Diabetic Retinopathy

RSV supplementation in diabetic rats has demonstrated a significant improvement in the symptoms of hyperglycaemia [83]. Treatment using RSV has been shown to inhibit diabetic changes like increased vascular leakage and loss of pericytes while it facilitates regulation of the VEGF (vascular endothelial growth factor) protein levels in mouse retinas [84]. Li et al. demonstrated that RSV might reduce endoplasmic reticulum stress, which contributes significantly to retinal vascular degeneration [85]. The study showed that RSV-treated cells significantly inhibited the accumulation of VEGF, TGF-β1 (transforming growth factor -β1), COX-2, IL-6, and IL-8 in a dose-dependent manner. Furthermore, the activity of the kinase C-beta protein (PKCβ) was also significantly reduced when presented with RSV. It is known that PKCβ regulates the activity of VEGF under hypoxic conditions, which additionally contributes to the degradation of the blood-retinal barrier [86]. Hua et al. investigated the development of retinal neovascularization and found that RSV ingestion in mice, starting five days before the neovascularization first appeared, reduced all lesion development observed in the retina by 70% after 30 days [87].

### 3.2. Glaucoma

The way RSV influences the expression of glaucoma markers in cells of the trabecular reticulum subjected to chronic oxidative stress was investigated [2,88]. Oxidative stress is a known factor that contributes to altering the function of the trabecular reticulum. This alteration leads to disturbances in circulation in the aqueous humour and ultimately to ocular hypertension. The study has demonstrated that RSV effectively reduced the production of intracellular ROS, decreased inflammatory markers of interleukins IL-1α, IL-6, IL-8, and limited the production of the endothelial-leukocyte adhesion molecule-1 (ELAM-1). RSV has been found to potentially play an anti-apoptotic role in trabecular reticulum cell damage prevention [88]. The study has shown that riluzole and resveratrol therapies exhibited neuroprotective properties in the survival of retinal ganglion cells (RGCs) in an experimental glaucoma model. Moreover, early treatment demonstrated more effectiveness than late treatment, as indicated by the RGCs densities for both factors. Moreover, the combined use of both drugs was associated with a significantly higher RGCs survival rate in comparison to riluzole or resveratrol alone [22].

### 3.3. Age-Related Macular Degeneration (AMD)

AMD is inextricably linked to retinal pigment epithelial (RPE) abnormalities [89] and can be classified as dry or wet. A characteristic feature of dry AMD is the accumulation of drusen (lipofuscin) around the RPE, accompanied by some degree of RPE degeneration [89]. Wet AMD choroidal neovascularization (CNV) is present, and newly formed blood vessels proliferate into the RPE or subretinal space [89]. Mounting evidence suggests that RPE injury and death due to a variety of mechanisms, including inflammation and stress, play a role [90,91]. RSV has been shown to prevent apoptosis of human RPE cells in vitro [92]. Moreover, it has been demonstrated that RPE cell proliferation is reduced by inhibition of extracellular protein kinases regulated by the first and second signal (ERK 1/2) and the signaling cascade of mitogen-activated protein kinase (MAPK) [92,93]. RPE cells are also protected by RSV from autoimmune antibody-induced apoptosis in vitro, which is of high importance in autoimmune-related retinopathies [94]. Dose-related protective effect against cytotoxicity induced by hydrogen peroxide in human cells has been reported. This is due to an increase in superoxide dismutase, glutathione peroxidase, and catalase activity, which inhibits intracellular ROS [95]. Research suggests further benefits of the antioxidant effect of RSV supplementation, such as preventing oxidative stress-induced degeneration of the RPE. Choroidal angiogenesis often characterizes the late stages of AMD. It is believed that the anti-inflammatory and antioxidant properties of RSV reduce the incidence of CNV. The inhibitory effects of resveratrol on inflammatory cytokine, TGF-β, and hypoxia-induced VEGF secretion by human RPE cells have been investigated by Nagineni et al. The goal was to demonstrate resveratrol’s usefulness as a nutraceutical supplement that may control CNV processes in AMD [96]. Their study has shown that the suppression of inflammatory cytokine mix, TGF-β, and hypoxia enhanced VEGF-A and VEGF-C secretion by human RPE without influencing anti-angiogenic endostatin and pigment epithelial-derived factor secretion by resveratrol. Moreover, VEGF secretion was inhibited by resveratrol down-regulated expression of NFκB and hypoxia-induced factor (HIF)-1α transcription factors. An important modulator of resveratrol—SIRT1 represses HIF-1α signaling due to deacetylation, which leads to a reduction in VEGF-A secretion [97]. It has also been shown that resveratrol acts by activating eukaryotic elongation factor-2 (eEF2) kinase and suppresses VEGF secretion. It also inhibits endothelial cell proliferation and migration by a novel SIRT1 independent pathway. This prevents pathologically aberrant angiogenesis induced by injury [98]. A report on nutritional supplementation in AMD found that a short-term RSV effect resembles the effects of anti-VEGF treatment with the anatomical restoration of retinal structure, RPE function improvement, and choroidal blood flow [80].

### 3.4. Cataract

RSV has been proven to inhibit the formation of selenite-induced cataracts due to stimulation of increased levels of reduced glutathione (GSH) and reducing levels of malondialdehyde (MDA), which is a lipid peroxidation marker in rat lenses [99]. A study conducted on human lens epithelial cells has shown that RSV reduces cell death and ROS accumulation following an acute state of H_2_O_2_ oxidative stress. This protective effect in cells is due to the increased expression of enzymes such as superoxide dismutase-1 (SOD-1), catalase, and heme oxygenase-1 (HO-1) [100]. In diabetic cataract pathogenesis, high glucose levels induce oxidative damage in human lens epithelial cells (HLEC). The development of pathogenic diabetic cataracts is associated with inappropriate metabolic regulation [101]. The accumulation of sorbitol produced in enzymatic glucose reduction by aldose reductase has been established as an initiating factor in the pathogenesis of diabetic cataracts. RSV has been shown to be effective in preventing and treating diabetic cataracts, as well as suppressing high glucose-induced oxidative damage by activating p38 MAPK signaling to promote autophagy in HLECs [101]. These results suggest that RSV can be utilized in the prevention and treatment of diabetic cataracts [101]. Other researchers have reached similar results [102]. Posterior capsule opacification has been shown to be possibly inhibited by RSV after cataract surgery [103].

### 3.5. Uveitis

Kubota et al. have studied the effects of RSV use in uveitis. In order to assess the protective effect of resveratrol, uveitis was induced with endotoxin in a mouse model. This study has demonstrated that oral RSV supplementation for five days of prophylaxis inhibited the production of the following proteins, which are key in the inflammatory process: Inter Cellular Adhesion Molecule-1 (ICAM-1) and Monocyte Chemoattractant Protein 1 (MCP-1) [104].

### 3.6. Eye Tumors

RSV treatment results in death and regression of neoplastic cells [105,106,107]. RSV suppresses the growth of uveal melanoma through early mitochondrial dysfunction and caspase-3 activation [105]. It has been shown that RSV caused a 50% inhibition of tumor growth throughout a 3 to 5 week period at drug doses from 2 to 50 mg/kg [105]. Sareen et al. demonstrated that due to RSV, the growth of the retinoblastoma cell line in a time- and dose-dependent manner is inhibited, while cycle S-phase cell arrest and apoptosis are promoted [106]. Other researchers reached similar results [107].

### 3.7. Retinopathy of Prematurity (ROP)

Kim et al. were able to demonstrate the effectiveness of RSV in the treatment of oxygen-induced retinopathy in rats, which may point to a protective role in ROP in premature infants [108]. Both in vivo and in vitro expression of iNOS (inducible nitric oxide synthase) antibodies and mRNA was increased, while eNOS (endothelial NOS) and nNOS (neuronal NOS) decreased in the group treated with resveratrol [108]. Li et al. found that treatment with RSV significantly inhibited Bcl-2 (B-cell lymphoma 2) and VEGF expression in the retina of neonatal rats with oxygen-induced ROP [109].

### 3.8. Corneal Infections and Neovascularization

There was a study performed in vivo which showed that RSV reduces the cytotoxicity of Acanthamoeba castellanii in the HBMEC cell line [110]. Marino et al. demonstrated that RSV might positively affect the course of Staphylococcus aureus keratitis in an ex vivo culture model of rabbit cornea [111]. The antioxidant action of RSV on a human epithelial cell line of the cornea exposed to levofloxacin (moxifloxacin) was researched by Tsai et al. In their study, RSV increased the chances of cell survival and reduced oxidative stress and its intracellular accumulation [112]. There are contradictory studies on the influence of RSV on the course of corneal neovascularization. Brakenhielm et al. showed that the use of RSV had a beneficial effect on the course of corneal neovascularization in mice. They induced angiogenesis by implanting corneal tissue impregnated with VEGF and type 2 fibroblast growth factor (FGF-2). The appearance of dense neovascularization around the periphery of the cornea was the result of this. The three-day intake of resveratrol in drinking water prior to implantation reduced the area of VEGF and FGF-2-induced neovascularization and the density of vascularization [113]. On the other hand, Doganay et al. found no effect of RSV supplementation on corneal neovascularization in the experimental rabbit models [114].

### 3.9. Dry Eye Disease (DED)

Vitamin D deficiency may be one of the factors responsible for the development of DED. Supplementation of this vitamin alleviates the symptoms of the disease. In hyperosmolar conditions, RES regulates vitamin D receptors most likely through Notch signaling activation. Thus, RES may potentially be an adjuvant in DED patients considered for vitamin D treatment [115]. A beneficial anti-inflammatory effect of RSV has also been demonstrated in patients with dry eye disease [116]. In RSV-treated DED mice, lower levels of IL-1 and CD4 + T cells were found in tears [116].

### 3.10. Vision Defects—Myopia

RSV supplementation may benefit those suffering from myopia. Various factors have been shown to contribute to the development of myopia, including TGF-β, matrix metalloproteinase-2 (MMP2), and collagen I. TGF-β is expressed in tissues of the eye and remodels the scleral extracellular matrix (ECM) [117,118,119], which reduces collagen production. Collagen disorders are exacerbated by the activation of the nuclear factor-κB, which increases the expression of matrix metalloproteinase 2 while reducing the activity of the tissue MMP2 inhibitor [120]. Hsu et al. found shorter eyes in hamsters treated with RSV than in the group that had not been treated with RSV [121]. A decrease in resveratrol-treated eyes has been observed in myopia-related tissue remodeling proteins and the expression of inflammatory cytokines (NFκB, TGF-β, MMP2, TNFα, IL-6, and IL-1β). In addition, collagen I expression increase in eyes treated with resveratrol was noted. This might suggest that resveratrol exhibits a regulation pattern similar to atropine [121].

### 3.11. Vitreoretinopathy

The development of PVR relies on other actors, such as the migration and proliferation of RPE cells, glial cells, and inflammatory cells. It has been demonstrated that RSV inhibits the development of vitreoretinopathy. RSV also suppresses the drop-off in zona occludens-1 and results in an increase in alpha-smooth muscle actin expression in TGF-β2-treated cells. RSV decreases TGF-β2-induced wound closure and cell migration. It also reduces the contraction of collagen gel. RSV inhibits PDGF-BB, an isoform of PDGF, and PI3K/Akt, ERK, and p38 pathways [11].

## 4. Resveratrol Supplementation

Grapes, wine, apples, peanuts, and soybeans are believed to be the main dietary sources of RSV. RSV has also been found in blueberries and cranberries [122,123]. As the concentration of RSV in food products is variable, it is difficult to estimate the average daily consumption [6]. Typical resveratrol concentrations reported for conventional food products are shown in Table 1 [6,124].

Resveratrol acquired from the *Polygonum cuspidatum* (Fallopia japonica, Japanese knotweed) root extracts, red wine, and grape (*Vitis vinifera*) extracts are widely available as dietary supplements. Various supplement tablets or capsules contain doses of resveratrol ranging from less than 1 milligram (mg) to 500 mg [2]. Resveratrol bioavailability exhibits a high inter-individual variability independent of age and gender [125]. The recommended daily dose of RSV is equivalent to 12.5 mg/kg of body weight, assuming an adult weighs 80 kg. These concentrations were based on animal studies. Most require daily doses of 5–100 mg of resveratrol/kg of body weight to achieve a specific biological effect [6]. Thus, according to Weiskirchen et al., it is not possible to absorb the recommended dose of RSV by taking any of these nutrients or their combination [6]. Pharmacokinetic studies of trans-resveratrol in humans have shown the serum concentration of unmetabolized RSV to be very low, both on oral and intraperitoneal administration [2]. RSV appears to be absorbed easily (~75%) on oral administration in humans, mainly via transepithelial diffusion, yet its bioavailability is relatively low (<1%). This is because it is metabolized rapidly in the intestine and liver [126]. Studies on increasing doses from 25 to 5000 mg of RSV have not shown the expected linear increase in plasma RSV concentration [19]. Even after the highest dose (5000 mg), peak plasma levels only reached about 500 ng/mL. Consumption of low doses of RSV has been shown to produce peak plasma concentrations within the first 30 min, while a high dose of RSV has been shown to produce peak plasma concentrations after 1.5–2 h [127]. Rapid absorption, poor bioavailability, and low water solubility are the main limitations and challenges of in vivo use of RSV [128,129]. In order to improve these parameters, attempts were made to use solid lipid nanoparticles (SLN) and nanostructured lipid carriers (NLC) [130]. RSV metabolites were also investigated [131]. Three metabolic pathways of RSV have been discovered—conjugation of phenolic groups with sulfate and glucuronic acid and hydrogenation of the aliphatic double bond produced by the intestinal microflora—dihydroresveratrol [128]. RSV analogs, such as methylated derivatives with improved bioavailability, may be an important subject of future research [126]. Piceid is the glycosylation product of RSV and the main form of stilbene storage in nutrients. Administering piceid may be an alternative to consuming pure compounds. Burkon and Somoza suggested that piceid may be enzymatically hydrolyzed in the colon or inside the enterocytes, resulting in the formation of trans-RSV. Therefore, piceid may be an alternative form of administration of soluble RSV [132].

RSV is generally considered safe [6]. No RSV toxicity has been reported in studies ranging from 14 days to 3 months of RSV administration and a dose range of 15 mg to 5 g [127]. There are studies that reported mild adverse effects such as headaches, dizziness, epididymitis, nausea, diarrhea, and abdominal discomfort, but only at higher doses (2.5 and 5.0 g) [6,127,133]. Other adverse effects reported at 5 g of micronized resveratrol (SRT501) include chills, lethargy, rash, skin irritation, and vascular flushing [2,134]. RSV supplementation is not recommended during pregnancy. Studies carried out on monkeys demonstrated that a diet enriched with RSV has a positive effect on improving glucose tolerance, increases blood flow to the fetus, or reduces inflammation of the placenta and liver. However, pancreas enlargement has been found in fetuses [135]. Most of the research on RSV has been done on animals. Its mechanism of action and optimal doses in humans may differ [81]. Moreover, women with a history of estrogen-sensitive cancer should avoid resveratrol supplements until there is no information about the estrogenic activity of resveratrol in humans [2,136]. There is no information on the lethal dose of RSV in all the available literature. Additionally, the range of concentrations at which RSV exceeds the desired effect has not been established [81]. Given the poor oral availability, it may be important to identify other preferred routes for RSV administration. There are reports that local injections of RSV around tumors had a better effect on the reduction of its mass than oral administration [105].

RSV may reduce iron absorption [137,138]. Therefore, those suffering from anemia should not supplement RSV. It is important to consider the interactions of RSV with other drugs. RSV may inhibit human platelet aggregation in vitro [2]. Theoretically, high consumption of RSV may therefore increase the risk of bruising and bleeding when taken with anticoagulant, antiplatelet, and non-steroidal anti-inflammatory drugs [2]. It is also believed that caution should be exercised with the use of RSV and the use of antihypertensive drugs (calcium channel blockers, e.g., nifedipine), statins, antihistamines, psychotropic drugs (e.g., triazolam), immunosuppressants, antifungal drugs, and erectile dysfunction medication [139].

## 5. Conclusions

Due to its wide range of therapeutic effects, RSV can be used in many fields of medicine, ophthalmology among them. Numerous in vivo and in vitro studies have been conducted to demonstrate its effectiveness in a variety of eye diseases. Undoubtedly, further research is needed to fully understand the mechanisms of RSV activity. Due to promising results, RSV may be implemented in the diet, although its limited systemic availability may be considered a major disadvantage. Rapid absorption and metabolism of RSV are important factors that limit its effectiveness. Further clinical trials will be necessary before any recommendations can be made regarding the use of RSV as an eye health supplement. Hopefully, in the years to come, ways will be found to improve them and make use of the full potential of resveratrol.

## Figures and Tables

**Table 1:** Resveratrol concentration in food products.

Food Products	RSV Concentration
peanuts without seed coats,	0.03–0.14 μg/g
red wines	0.361–1.972 mg/L
white wines	0–1.089 mg/L
rosé wines	0.29 mg/L
beers	1.34–77.0 μg/L
skin of tomatoes	∼19 μg/g dry weight
dark chocolate	350 μg/kg;
milk chocolate	100 μg/kg
Itadori tea	68 μg/100 mL
red grapes	92–1604 μg/kg fresh weight
white grapes	59–1759 μg/kg fresh weight
apples	400 μg/kg fresh weight
Cranberry raw juice	~0.2 µg/mL
Blueberries	Up to ~0.032 µg/g
Bilberries	Up to ~0.016 µg/g
Boiled peanuts	5.1 µg/g
100% Natural peanut butters	0.65 µg/g (average)
Polygonum cuspidatum herb	524 µg/g

RSV: Resveratrol.
